# Enhancing classification of preterm-term birth using continuous wavelet transform and entropy-based methods of electrohysterogram signals

**DOI:** 10.3389/fendo.2022.1035615

**Published:** 2023-01-10

**Authors:** Héctor Romero-Morales, Jenny Noemí Muñoz-Montes de Oca, Rodrigo Mora-Martínez, Yecid Mina-Paz, José Javier Reyes-Lagos

**Affiliations:** ^1^ Interdisciplinary Unit of Biotechnology (UPIBI), National Polytechnic Institute (IPN) of Mexico, Mexico City, Mexico; ^2^ National Institute of Astrophysics, Optics and Electronics (INAOE), Tonantzintla, Puebla, Mexico; ^3^ Health and Movement Research Group, Faculty of Health, Universidad Santiago de Cali, Cali, Colombia; ^4^ School of Medicine, Autonomous University of the State of Mexico (UAEMéx), Toluca de Lerdo, State of Mexico, Mexico

**Keywords:** electrohysterography, entropy, time-frequency analysis, uterine electromyogram, preterm labor, machine learning

## Abstract

**Introduction:**

Despite vast research, premature birth's electrophysiological mechanisms are not fully understood. Prediction of preterm birth contributes to child survival by providing timely and skilled care to both mother and child. Electrohysterography is an affordable, noninvasive technique that has been highly sensitive in diagnosing preterm labor. This study aimed to choose the more appropriate combination of characteristics, such as electrode channel and bandwidth, as well as those linear, time-frequency, and nonlinear features of the electrohysterogram (EHG) for predicting preterm birth using classifiers.

**Methods:**

We analyzed two open-access datasets of 30 minutes of EHG obtained in regular checkups of women around 31 weeks of pregnancy who experienced premature labor (P) and term labor (T). The current approach filtered the raw EHGs in three relevant frequency subbands (0.3–1 Hz, 1–2 Hz, and 2–3Hz). The EHG time series were then segmented to create 120-second windows, from which individual characteristics were calculated. The linear, time-frequency, and nonlinear indices of EHG of each combination (channel-filter) were fed to different classifiers using feature selection techniques.

**Results:**

The best performance, i.e., 88.52% accuracy, 83.83% sensitivity, and 93.22% specificity, was obtained in the 2–3 Hz bands using Medium Frequency, Continuous Wavelet Transform (CWT), and entropy-based indices. Interestingly, CWT features were significantly different in all filter-channel combinations. The proposed study uses small samples of EHG signals to diagnose preterm birth accurately, showing their potential application in the clinical environment.

**Discussion:**

Our results suggest that CWT and novel entropy-based features of EHG could be suitable descriptors for analyzing and understanding the complex nature of preterm labor mechanisms.

## 1 Introduction

Preterm birth (PB), which affects 15 million newborns worldwide, is the leading cause of neonatal mortality and morbidity (1). PB is considered a multifactorial syndrome associated with rupture of membranes, uterine abnormalities, infections, and multifetal pregnancy (2). Prevention of PB includes a set of measures taken in early-stage patients to stop or delay its effects, which is why an early diagnosis is essential in developing a patient-oriented care strategy and reducing neonatal death. However, current diagnostic tools of PB are limited to algorithms for predicting risk based on the patient’s clinical history, signs, and symptoms instead of focusing on the syndrome’s underlying mechanisms.

The progress of labor can be monitored by a tocodynamometer, which lacks sensitivity to detect slight uterine activity, or by an intrauterine pressure catheter, an invasive technique for diagnosing PB (3). Electrohysterography is a noninvasive technique that uses surface electrodes in the mother’s abdomen to obtain electrical information about the activity of myometrial cells by quantifying uterine action potentials (3). This technique is sensitive to properly detect uterine electrical activity, which allows continuous monitoring the progress of labor and detection of dystocias. Notably, relevant evidence revealed that electrohysterography is more sensitive than external tocodynamometry in detecting uterine contractions during the early stage of labor (4). Various features of the uterine electromyogram or electrohysterogram (EHG) have been continuously studied to differentiate between preterm and term birth (3). Interestingly, the EHG is advised for further introduction and testing in clinical practice based on recent research that suggests that it has no adverse effects (5).

Despite ongoing research on EHG, predicting premature birth from it remains a complicated problem. One of the main difficulties arises at the signal preprocessing stage, where there is still a recurrent debate about where the predominant spectral contents of the EHG are located (6, 7). In addition, the pool of attributes that describes more appropriately the electrophysiological phenomena of preterm birth and the classifier type is still an object of continuous study to develop an accurate prediction tool (6–9). Previous studies have explored different methods to automatically classify EHG signals and diagnose preterm birth (**Table 1**). According to these results, the combination of time-frequency analysis with nonlinear signal indices of EHG may enhance the classification of preterm-term births. Wavelets can capture subtle variations in transient signals using time-frequency analysis approaches and nonlinear signal processing techniques (11).

**Table 1:** Summary of previous studies for the prediction of preterm birth by obtaining linear, time-frequency, and nonlinear EHG features.

Authors	Classifier type	Dataset	Length	Features	Accuracy	Sensitivity	Specificity	AUC
Acharya et al., (2017) (6)	Support vector machine (SVM) Radial Basis Function (RBF)	TPEHG DB (34 recordings of preterm and 262 of the term)	Complete recording	Empirical Mode Decomposition (EMD) Wavelet Packet Decomposition (WPD) Entropy-based methods	96.25	95.08	97.33	0.96
Jager et al., (2018) (7)	Quadratic Discriminant	TPEHG DB TPEHG DS	Complete recording	Sample Entropy Medium Frequency Maximum Frequency	100.00	100.00	100.00	1.00
Nieto del Amor et al., (2021) (9)	Linear Discriminant	TPEHG DB	120-seconds window Complete recording	Dominant frequency Normalized Energy Power spectrum deciles (D3, D6, D8, D9) Entropy-based methods	89.2% ± 2.4	98.4% ± 1.9	79.9% ± 4.9	0.93
Hoseinzadeh & Amirani (2018) (10)	RBF SVM	TPEHG DB	N/A	EMD WPD Feature extraction by autoregressive models	97.1	95	99	N/A

N/A, Not available.

The present study aims to expand the setlist of time-frequency and nonlinear indices of EHG aiming to improve the predictive accuracy of current classifiers for predicting preterm birth. Thus, novel parameters such as Flux and the Energy derived from the Continuous Wavelet Transform (CWT) and multiple scales of Phase Entropy were analyzed. To our knowledge, these characteristics have not been tested for the differentiation between term and preterm labor; however, previous studies have shown their adequacy in the characterization of physiological signals in pregnant women (12, 13).

This study also attempted to select the best combination of electrode channel, bandwidth, pool of linear and nonlinear characteristics of the EHG, and type of classifier, to achieve a predictive accuracy higher than 85%. We consider that developing a robust model for preterm labor prediction would offer an advance in the diagnosis and timely treatment of the population at risk, thereby reducing neonatal mortality and contributing to the application of EHG in the clinical setting.

## 2 Materials and methods

**Figure 1:**
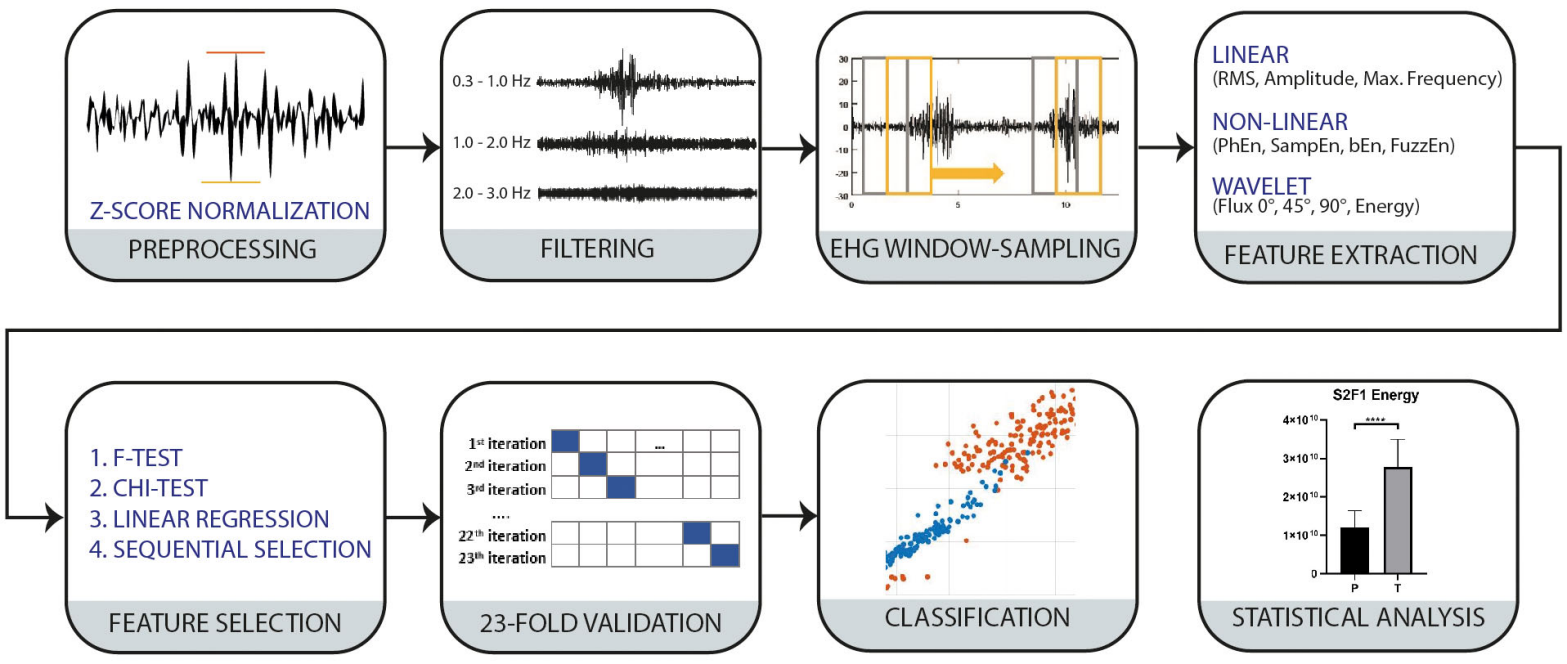
Block diagram of the proposed methodology for P and T classification.

The proposed methodology is presented in **Figure 1**. It was divided into seven procedures that are furtherly explained in this section.

### 2.1 Dataset description

The data analyzed in the present study to differentiate between Preterm (P) and Term (T) groups were obtained from the open-access databases Term-Preterm EHG DataSet with Tocogram (TPEHG DS) and the Term-Preterm EHG DataBase (TPEHG DB) available on the Physionet website (7, 14, 15). The TPEHG DS includes 26 EHG signals recorded at the University Medical Center Ljubljana, Department of Obstetrics and Gynecology. Twelve EHG recordings of the P group were collected from 8 healthy subjects whose pregnancy ended at 33.7 ± 1.97 weeks of gestation (WG). The T group comprises 13 EHG recordings of ten healthy participants whose labor onset triggered around 38.1 ± 1.04 WG. Thus, the data analyzed for both T and P conditions is formed by 30 minutes of raw EHG obtained in regular medical checkups around the 31^st^ (30.2 ± 2.76) WG. Similarly, the TPEHG DB consists of 300 records, 262 T and 38 P, obtained from 1997 to 2005 at the University Medical Center Ljubljana. 17 records from each group (P and T) were selected to test the classification model to obtain a balanced dataset. Data were selected based on gestational age (between the 27^th^ and 33^rd^ WG) to maintain time compatibility with the TPEHG DS. The P group contains only 17 records that match our inclusion criteria. These were taken from healthy participants who delivered around 34.7 ± 2.02 WG. From the T group, 119 records were obtained during or after the 26^th^ week of gestation. However, to maintain a balanced dataset, 17 were randomly selected. These records belong to 17 healthy patients whose labor triggered around 39.3 ± 1.36 WG.

**Figure 2:**
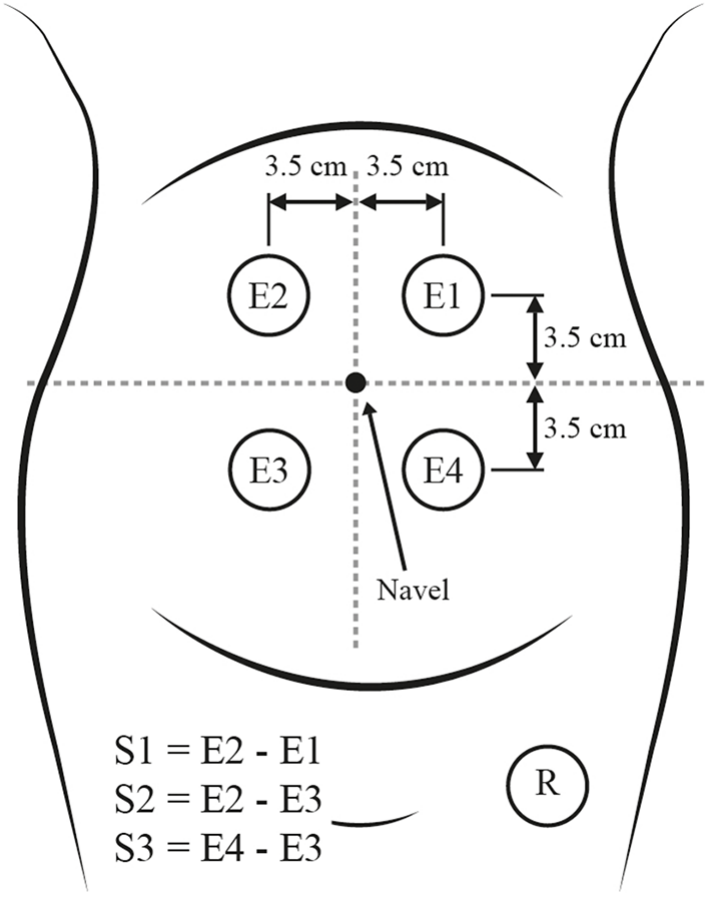
Placement of electrodes in the maternal abdomen (modified from 7).

The EHG signals from the TPEHG DS and TPEHG DB datasets were acquired using four AgCl_2_ electrodes positioned on the abdominal surface in a 2 x 2 matrix configuration, placed symmetrically above and under the navel with a space of 7 cm between each electrode, as shown in **Figure 2**. The reference electrode was placed in the mother’s thigh. Channels S1, S2, and S3 are bipolar signals resulting from the difference in potential of electrodes (E1, E2, E3, and E4), as stated in **Figure 2**. Each signal was digitalized at 20 samples per second with a 16-bit resolution.

In this study, the signal p001 corresponding to P was discarded by a visual examination, given that motion artifacts possibly corrupted the signal in E1 and did not correlate with the tocogram’s simultaneous acquisition.

### 2.2 Signal preprocessing

While there is no consensus on the frequency range of the EHG signal, several authors suggest that the main frequency content is in the frequency band of 0.1 – 4.0 Hz (3, 7). The EHG is often divided into two frequency subbands: Fast Wave Low (0.1 – 1.2 Hz), associated with the propagation of the electrical signal, and Fast Wave High (1.2 – 4.7 Hz), associated with myometrial excitability. Given that there is no convention about the EHG bandwidth, three pass-band IIR filters were applied to each raw EHG channel in the following subbands: F1 (0.3 – 1 Hz), F2 (1 – 2 Hz), and F3 (2 – 3 Hz) conducting the procedure suggested by Selvaraju et al. (16). This filtering step evaluated the best frequency content to classify between P and T groups. In addition, it is also suggested that F1 holds valuable information related to myometrial cell activity; at the same time, bands F2 and F3, altered by maternal electrocardiogram (ECG) interference and its harmonics, may be relevant biomarkers to evaluate preterm birth (7). Preprocessing of the signal also included an amplitude standardization using z-score normalization. This method was applied to create homogenous conditions for data processing between the complementary datasets employed.

Several studies have obtained promising results in predicting preterm birth by isolating contractions or bursts from the EHG records (11, 17–19). However, that methodology requires the supervision of qualified personnel or the simultaneous use of tocodynamometry (TOCO). In this study, 120-second windows with a 50% overlap were extracted from the filtered EHG records, according to Nieto del Amor et al. (9). Nevertheless, the present study differs from that proposed in (9) by considering each 120-second window as a repeated measurement for predicting preterm birth. Window extraction was performed automatically, overcoming the disadvantages and time-consuming procedures of EHG-burst identification. Its simplicity and short length could make this algorithm suitable for its implementation in a clinical environment.

Prior to window sampling, the EHG data was randomly and uniformly split into training (70%), validation (15%), and testing (15%). This procedure was done for the complete record to avoid model bias. Thus, the training, testing, and validating groups contain information from independent recordings.

Thirty-three features were calculated for each EHG window, including linear: Maximum and Medium Frequency, Root Mean Square (RMS), Amplitude, Zero Crossing Rate (ZCR), and nonlinear: Sample Entropy (SampEn), Fuzzy Entropy (FuzzEn), Permutation Entropy (PerEn), Bubble Entropy (bEn), and Phase Entropy (PhEn). Specifically, novel characteristics such as PhEn and Continuous Wavelet Transform (CWT) derived features (Energy and Flux on 0°, 45°, and 90° used to evaluate cardiotocography signals (12)) were introduced to identify preterm labor. The CWT was calculated by the continuous 1-D wavelet transform, using the analytic Morse wavelet, with a symmetry parameter equal to 3 and L1 normalization, ensuring an equal signal representation (20). Additionally, DWT coefficients were calculated using the methodology proposed by the author Janjarasitt, using the ‘Daubechies wavelet’ of 12^th^ order, decomposing the signal in 7 levels, and calculating the difference between adjacent level coefficients (8). **Table 2** depicts the set of parameters calculated for each EHG segment.

**Table 2:** List of linear, nonlinear, and time-frequency features extracted from electrohysterographic signals.

Linear	Nonlinear (Entropy-based)	Time-frequency (Wavelet)
Maximum Frequency (21)	Phase Entropy (PhEn) (22)	Flux 0° (12)
Medium Frequency (21)	Sample Entropy (SampEn) (23)	Flux 45° (12)
Root Mean Square (RMS) (24)	Dispersion Entropy (DispEn) (25, 26)	Flux 90° (12)
Zero-Crossing Rate (ZCR) (27)	Permutation Entropy (PerEn) (28, 29)	Energy (12)
Amplitude (21)	Bubble Entropy (bEn) (30, 31)	DWT (8)
	Fuzzy Entropy (FuzEn) (32)	

The entropy-based features calculated in the present study possess internal input values that modify the discriminating power between the classes P and T. Past studies have focused on analyzing these values, finding the optimal combination of parameters to predict preterm birth (**Table 3**). In this work, we attempted to use those values to obtain an accurate predictive model and identify the most relevant characteristics to detect preterm birth using various entropy-based features.

**Table 3:** Internal parameters employed in the calculation of entropy and wavelet-based features.

Feature	Internal parameters
Sample Entropy	*m*=2, *r*=0.15
Phase Entropy	*k*=2,4,6,8,10,12,14,16,18,20,22,24
Permutation Entropy	*d*=2, *π*=3
Dispersion Entropy	*m*=2, *c*=3, linear mapping
Fuzzy Entropy	*m*=2, *r*=0.0077, *n*=3 exponential function, local and global
Flux (Continuous Wavelet Transform features) (k_1_, k_2_)	0°: (0,1); 45°: (1,1); 90°: (1,0)
Discrete Wavelet Transform analysis	12-order ‘Daubechies’ wavelet

The entropy-based feature of Permutation Entropy (PerEn) had not been used before in studying EHG signals; that is why we selected the input parameters *d* and *π* for PerEn according to previous suggestions for electromyography analysis (33). The internal parameter *k* was analyzed within this study for the novel entropy of PhEn; it has been previously explored for the differentiation between the third trimester and active parturition and between eutocic delivery and c-section but not for preterm birth detection (13, 34). The internal input parameter *k* was modified from 2–24, with a 2-step increase, generating twelve PhEn values, considered as individual characteristics for the algorithms of feature selection and the classifier design.

### 2.3 Feature selection and classifier design

We computed thirty-three features for each 120-second window of EHG in the subbands F1, F2, and F3 and for the three channels S1, S2, and S3. The classification models used in the current study are decision trees, support vector machine (SVM), and discriminant analysis.

Four feature selection algorithms (F-test, chi-test, linear regression, and sequential selection) were used to select the best features for each classification model, reducing the computational cost and increasing the classifier’s performance. Feature selection decreases the number of input variables by selecting characteristics that show a relevant relationship between the input and target variables (35). These methods result in a list of predictors in order of importance. Multiple runs for each algorithm resulted in a different subset of relevant features, and multiple selection algorithms were employed simultaneously to obtain consistent results. The comparative analysis among algorithms choices allowed us to select features better suited to describe preterm birth. In this comparative methodology, the features were selected as follows: the first ten features from the F-test and chi-test were selected. Linear regression computes the regression model for output and input variables and returns information on the statistical correlation of variables. Features with a significant p-value (*p*<0.05) were selected from the linear regression model. These features were sorted in ascending order, selecting the first ten results for the classifier. Sequential selection features are extracted by adding and sequentially extracting parameters until a condition is met and the prediction algorithm cannot be improved. Feature selection comprises the repeated characteristics within the tests employed: sequential selection features were compared to the other three models (F-test, chi-test, linear regression) the repeated features were selected. Then, the ten predictors selected from the linear regression were compared to the F-test and chi-test; these features were also employed.

Each predictive model was composed of a range from 7 to 13 characteristics, selected independently employing feature selection. Each dataset’s classifier type was trained through the Classification Learner Toolbox in Matlab v2020a (MathWorks, USA), using only the training set. The best classifier type was selected based on accuracy and AUC (Area Under the Curve) from the trained classifiers. This information was then used to perform K-fold cross-validation for each dataset.

### 2.4 K-fold cross-validation

K-fold cross-validation is a procedure used in machine learning for small datasets by dividing the data into three groups (training, validation, and test). It results in a less biased model since it ensures that every observation from the original dataset appears in the training and testing set (36).

In this type of cross-validation, the total samples of train and validation groups are randomly split into a *k* number of folds of equal sizes. Then a classifier model is generated *k* times, each taking a different fold as testing and the rest as training. In this study, a *k* with a value of 23 was employed, with each partition containing 49 samples, to include the whole dataset. The test group, which contains independent recordings not employed in the training or validation sets, is tested for each classification model created.

Parameters such as accuracy, sensitivity, specificity, Negative Predictive Value (NPV), Positive Predictive Value (PPV), Area Under the Curve ROC (AUC), recall, precision, and F-Score were calculated simultaneously during the cross-validation to evaluate the performance of each channel-filter configuration. All computations were performed in Matlab v2020a.

### 2.5 Statistical analysis

A statistical analysis of the selected features was performed to evaluate the changes in the mean values of linear, nonlinear, and time-frequency indices between the P and T conditions for all combinations of filter-channel. These comparisons were performed using a nonparametric Mann-Whitney test, considering *p*<0.05 as a significant difference. The statistical analysis was accomplished using GraphPad Prism version 8.0.2 for Windows (GraphPad Software, La Jolla, CA, USA).

## 3 Results

**Figure 3:**
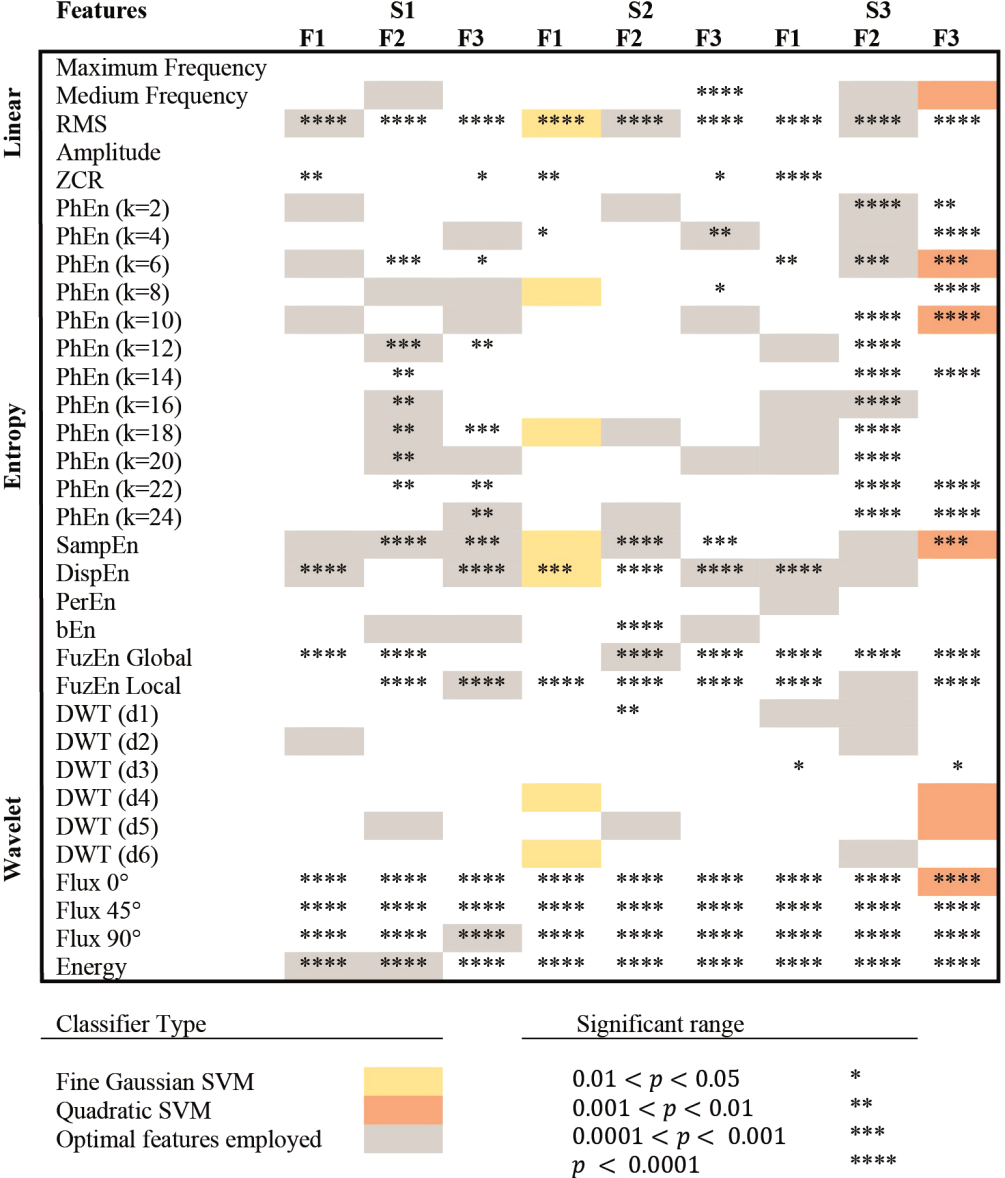
Selection of features for each classifier. S1, S2, and S3 represent the bipolar channels acquired from the public database, while F1, F2, and F3, represent the frequency sub-bands compared in this study. Colored frames (gray, yellow, and orange) indicate that the feature was selected as an optimal classification parameter and used to train the prediction model. The classifiers that achieved the highest classification accuracy (< 85%) in testing are presented in yellow and orange, indicating the type of classifier used. *,**,***,**** are used to describe significant differences between P and T groups in the Mann-Whitney test, with p-values lower than 0.05, 0.01, 0.001, and 0.0001, respectively.

Considering the alternative hypothesis that the EHG signals manifest differences between the T and P conditions, we independently analyzed each feature used within the classifiers. **Figure 3** shows the statistical analysis of all features employed in this study and indicates the features selected as optimal for classification and used to train the prediction models. The lowest p-values (*p*<0.0001) were found in the following features: linear (RMS), entropy-based indices (DispEn, SampEn, FuzzEn), and CWT-based features (Flux, Energy). It is also noticeable that the features of the PhEn were selected for all the models. Similarly, CWT features were employed in 4 out of 9 classifiers, including the best model (S3F3). Other entropy measures, such as DispEn, and SampEn, showed potential for classification. Linear Features of interest, RMS, and Medium Frequency, were also selected in various classifier models.

**Table 4** shows the higher classifier performances obtained from the k-fold cross-validation procedure from all classifier models. Notably, the classifier models of fine gaussian SVM in channel S2 within the 0.3 – 1 Hz subband (S2F1) and the quadratic SVM in channel 3 in the 2 – 3 Hz subband achieved an accuracy higher than 85%. The S3F3 model achieved an accuracy of 88.52±1.47%, a sensitivity of 83.83%±3.07%, a specificity of 93.22±1.31%, and subsequently, AUC=0.89±0.02 in the testing. Interestingly, using the SVM classifier model, other classifier models reached similar performances, such as S1F1 and S1F3.

**Table 4:** Summary of k-fold cross-validation for different classification models trained using the selected features of linear, nonlinear, and frequency indices of EHG.

	Classification models		Accuracy (%)	Sensitivity (%)	Specificity (%)	PPV (%)	NPV (%)	AUC	F-Score
S1F1	Quadratic Supportive Vector Machine (SVM)	Train	95.56 ± 0.25	94.72 ± 0.36	96.36 ± 0.42	96.16 ± 0.42	95.01 ± 0.32	0.94 ± 0.03	0.93 ± 0.03
		Validation	93.52 ± 3.24	92.97 ± 6.09	94.13 ± 5.64	93.99 ± 5.64	93.44 ± 5.37	0.94 ± 0.03	0.93 ± 0.03
		Test	82.04 ± 1.11	64.09 ± 2.21	100.00 ± 0.00	100.00 ± 0.00	73.59 ± 1.19	0.82 ± 0.01	0.78 ± 0.02
S1F2	Cubic SVM	Train	97.56 ± 0.29	97.21 ± 0.34	97.89 ± 0.35	97.79 ± 0.35	97.34 ± 0.32	0.93 ± 0.04	0.93 ± 0.05
		Validation	93.17 ± 4.25	92.56 ± 5.99	93.76 ± 5.33	93.51 ± 5.72	93.08 ± 5.69	0.93 ± 0.04	0.93 ± 0.05
		Test	55.04 ± 3.31	25.83 ± 3.76	84.26 ± 4.95	62.69 ± 9.33	53.17 ± 2.04	0.55 ± 0.03	0.36 ± 0.05
S1F3	Fine Gaussian SVM	Train	94.90 ± 0.170	94.63 ± 0.26	95.16 ± 0.31	94.94 ± 0.3	94.86 ± 0.22	0.92 ± 0.03	0.91 ± 0.04
		Validation	91.84 ± 2.75	91.22 ± 6.55	92.07 ± 4.70	91.90 ± 3.98	92.05 ± 4.55	0.92 ± 0.03	0.91 ± 0.04
		Test	81.13 ± 1.39	94.52 ± 1.24	67.74 ± 2.03	74.57 ± 1.30	92.52 ± 1.69	0.81 ± 0.01	0.83 ± 0.01
**S2F1**	**Fine Gaussian SVM**	**Train**	**96.63 ± 0.20**	**97.76 ± 0.25**	**95.55 ± 0.30**	**95.47 ± 0.29**	**97.80 ± 0.23**	**0.88 ± 0.04**	**0.88 ± 0.04**
		**Validation**	**88.20 ± 4.28**	**88.88 ± 5.85**	**88.04 ± 6.69**	**87.75 ± 6.95**	**88.99 ± 6.09**	**0.88 ± 0.04**	**0.88 ± 0.04**
		**Test**	**87.52 ± 1.20**	**75.04 ± 2.40**	**100.00 ± 0.00**	**100.00 ± 0.00**	**80.06 ± 1.54**	**0.88 ± 0.01**	**0.86 ± 0.02**
S2F2	Quadratic SVM	Train	91.70 ± 0.21	92.59 ± 0.38	90.84 ± 0.84	90.66 ± 0.33	92.74 ± 0.34	0.9 ± 0.04	0.9 ± 0.04
		Validation	90.33 ± 4.23	91.44 ± 5.84	89.41 ± 6.15	89.47 ± 5.40	91.07 ± 6.05	0.90 ± 0.04	0.90 ± 0.40
		Test	66.83 ± 1.03	37.57 ± 1.47	96.09 ± 2.05	90.82 ± 4.41	60.61 ± 0.61	0.67 ± 0.01	0.53 ± 0.01
S2F3	Weighted Kernel Nearest Neighbors (KNN)	Train	74.31 ± 0.44	70.34 ± 4.88	78.12 ± 4.62	75.82 ± 2.99	73.44 ± 1.97	0.68 ± 0.07	0.66 ± 0.08
		Validation	68.32 ± 3.490	64.16 ± 10.13	72.80 ± 9.32	69.28 ± 9.52	68.00 ± 9.51	0.68 ± 0.07	0.66 ± 0.08
		Test	51.04 ± 2.98	40.26 ± 5.16	61.83 ± 9.91	52.07 ± 4.65	50.65 ± 2.32	0.51 ± 0.01	0.45 ± 0.03
S3F1	Bagged Tree	Train	99.89 ± 0.09	99.93 ± 0.12	99.85 ± 0.16	99.84 ± 0.17	99.93 ± 0.12	0.71 ± 0.06	0.7 ± 0.09
		Validation	71.52 ± 6.2	68.9 ± 11.69	73.53 ± 9.78	71.35 ± 9.62	72.08 ± 5.98	0.71 ± 0.03	0.70 ± 0.09
		Test	47.91 ± 3.360	32.70 ± 3.60	63.13 ± 50	47.1 ± 4.61	48.36 ± 2.65	0.48 ± 0.03	0.39 ± 0.04
S3F2	Cubic SVM	Train	99.10 ± 0.22	99.3 ± 0.27	98.9 ± 0.29	98.86 ± 0.30	99.33 ± 0.12	0.93 ± 0.03	0.92 ± 0.04
		Validation	92.64 ± 3.18	93.16 ± 5.26	92.26 ± 4.56	91.73 ± 5.92	93.28 ± 5.02	0.93 ± 0.03	0.92 ± 0.04
		Test	72.17 ± 2.25	84.43 ± 1.59	59.91 ± 4.11	67.87 ± 2.30	79.35 ± 2.09	0.72 ± 0.02	0.75 ± 0.02
**S3F3**	**Quadratic SVM**	**Train**	**93.46 ± 0.31**	**93.57 ± 0.53**	**93.35 ± 0.44**	**93.11 ± 0.41**	**93.8 ± 0.48**	**0.91 ± 0.04**	**0.91 ± 0.04**
		**Validation**	**91.30 ± 3.65**	**92.10 ± 5.86**	**90.33 ± 5.14**	**90.37 ± 4.55**	**92.52 ± 5.31**	**0.91 ± 0.04**	**0.91 ± 0.04**
		**Test**	**88.52 ± 1.47**	**83.83 ± 3.07**	**93.22 ± 1.31**	**92.51 ± 1.46**	**85.27 ± 2.49**	**0.89 ± 0.02**	**0.88 ± 0.02**

The dataset employed for each classifier is denoted as S (1,2,3) for the signal channel and the frequency subband with F (1,2,3; 0.3 – 1 Hz, 1 – 2 Hz, 2 – 3 Hz, respectively). The highest classification accuracy is shown in bold.

**Figure 4:**
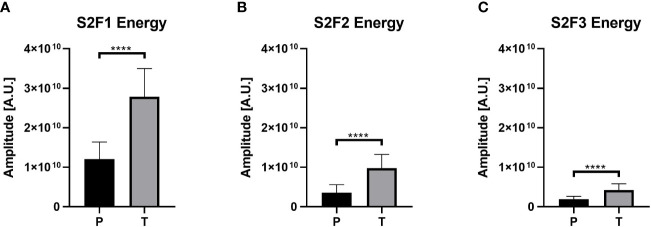
Energy values for the channel 2 in the three different subbands. The three panels can corroborate the difference between the P and T groups, where the T group presents higher values than P. **(A)** Values for the S2F1 combination. **(B)** Shows the values of Energy for S2F2 and **(C)** the Energy values for S2F3. **** represents p-value lower than 0.0001.

The statistical analysis results are shown in **Figure 3**, with the predictors used for each classifier. This figure highlights the low p-values of some relevant features, i.e., CWT characteristics and entropy-based methods. Interestingly, several significant differences (*p*<0.0001) in the mean values of CWT features such as Energy (**Figure 4**) were found between the T and P groups in the S2 channel: Energy of S2F1 (2.8x10^10^ ± 7.1x10^9^ amplitude in arbitrary units, A.U (Arbitrary Units) vs. 1.2x10^10^ ± 4.3x10^9^ A.U., **Figure 4A**); S2F2 (9.7x10^9^ ± 3.5x10^9^ A.U vs. 3.5x10^9^ ± 1.9x10^9^ A.U., **Figure 4B**) and S2F3 (4.2x10^9^ ± 1.6x10^9^ A.U vs. 1.8x10^9^ ± 7.6x10^8^ A.U., **Figure 4C**) for the P and T groups, respectively. In addition, the mean values of Flux from the spectrogram (0°, 45°, and 90°) were significantly higher (*p*<0.0001) in T compared to P conditions for the three subbands F1, F2, and F3 (data not shown).

**Figure 5:**
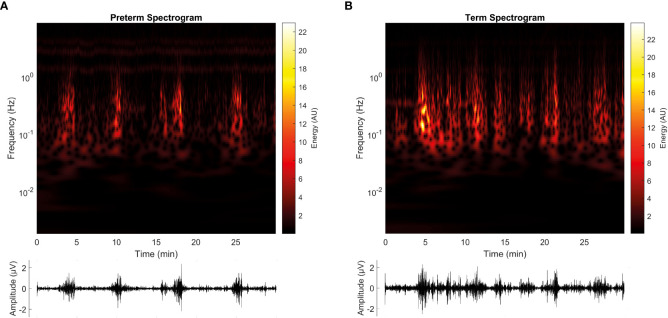
Representative spectrograms of EHG signals for the term (T) and preterm (P) groups. **(A)** shows the spectrogram retrieved from the participant no. 10, from the preterm group (P, recorded at 30 weeks of gestation and triggered labor at 32 weeks of gestation), while **(B)** shows the spectrogram retrieved from participant no. 9, from the term group (T, recorded at 30 weeks of gestation and triggered labor on 39 weeks of gestation). Below each spectrogram, the EHG signal can be visualized. Both spectrograms are derived from the S2 signal.

**Figure 5** depicts representative preterm (**Figure 5A**) and term (**Figure 5B**) participants’ spectrograms and their corresponding EHG. Interestingly, in these representative examples, the Energy of the T spectrogram is distributed on a broader frequency range and manifests a higher magnitude than P.

## 4 Discussion

Addressing physiological phenomena from an engineering interpretation can be challenging due to the prevailing gap between medical sciences and mathematics. However, it allows broadening the perspective of what is known about a topic. This work aimed to improve the general understanding of the mechanisms of parturition and preterm birth by applying different classifier models trained with EHG features such as RMS, CWT, and entropy-based methods. Therefore, nine classification models were designed to predict preterm labor, two of which achieved an accuracy higher than 85% using a k-fold cross-validation procedure.

Remarkably, our results showed that CWT-derived features are relevant in the characterization of EHG and could be suitable for implementing an algorithm to predict preterm birth. These results support the notion that the combination of metrics, such as linear, nonlinear, and even time-frequency features, complement each other for the purpose of classification (9). Thus, in addition to contributing to the classification performance, these new features also show relevant information on the physiological mechanisms of parturition. For example, RMS and CTW Energy both estimate the intensity of the womb’s electrical activity (7), and the spectrogram shows the Energy related to cellular activity or ‘bursts’ seen in the EHG signal (**Figure 5**).

The uterus comprises billions of intricately interconnected cells whose activity and responses are considered as a nonlinear dynamic process (37). Uterine electrical activity seems more irregular in the T compared to P labor. For example, PhEn values confirmed this behavior (T: 0.7973 ± 0.0372 vs. P: 0.7880 ± 0.0487)^1^ which is consistent with Reyes-Lagos et al., who compared EHG signals in the third trimester and parturition. That study showed a lower PhEn value for parturient women, which reflects the loss of irregularity of the EHG signal (13). Similarly, the high intensity, periodic and rhythmic contractions manifested at preterm labor (indicated by RMS, CWT Energy, and entropy-based measurements) differ from the term group, despite that the EHG signals were recorded around 31 weeks of gestation in both groups, which showed no clinical signs of labor. Evidence suggests that the accelerated development of gap junctions has been associated with preterm labor, typically resulting in better electric coupling and synchronization of myometrial activity (38).

The mechanisms involved in labor are still unknown for both term and preterm deliveries (39), which make difficult the research and clinical work to prevent preterm outcomes. However, a hypothesis having elevated level of acceptance postulates a series of biochemical, physiological, and anatomical changes occuring in labor. Nevertheless, it does not explain the central mechanisms, origin, or subsequent path of parturition. In this hypothesis, the onset of labor is assumed as a pro-inflammatory complex event triggered by a “decidual clock.” This is proposed as the mechanism that controls the initiation of delivery; however, it is still unclear how this clock works. According to this mechanism, the change between anti-inflammatory and pro-inflammatory mediators modifies the myometrium contractile state (39).

The parameters obtained by CWT analysis offer a new way to study EHG signals because they are based on the spectrogram that includes a three-variable visualization of the signal, incorporating time, frequency, and Energy. Jager et al. theorized that the shape of the uterus and cervix favors the influence of uterine muscle activity by propagating maternal heart rate electromechanically (7). Owing to the magnitude of EHG (500 uV), the electrical heart activity (1 mV) (26, 40) could be identified through EHG records (3). During gestation, the closed uterus reflects electrical pulses from the maternal electrocardiogram, causing interference. Thus, interference is expected to be more prominent in the T group (7). Furthermore, as labor progresses, cervical effacement generates an opening in the uterus, causing electrical signals to be diffracted, consequently the energy concentration is diminished in higher sub-bands (**Figure 4**).

In preterm spectrograms, the EHG signal is visually contained within the F1 frequency band (0.2–1 Hz), which is frequently related to EHG (5, 7, 9, 21). Sub-bands F2 and F3 are also identifiable, separated from the high-energy band. However, in term spectrograms, the EHG activity shows a a broader range that is translated to energetic components overlapping in bands F1, F2, and F3, as derived from the ECG interference. This idea is furtherly supported by CWT-derived features, in which the energy values in sub-band F2 (1–2 Hz) are higher for the T group (**Figure 4B**), where the interference derived from maternal heart activity is expected.

The Flux derived from CWT analysis measures the rate of change of local power in the time-frequency sphere (12). Flux was calculated at 0°, 45°, and 90°, establishing the direction of the signal’s propagation. A higher flux value represents higher power changes in the signal and direction. Diab et al. measured the directionality of a single EHG burst, demonstrating that during labor, the signal is propagated along the entire matrix of electrodes (41). However, their model also showed a tendency of propagation towards the cervix, generating efficient contractions to expel the fetus. Taking this into account, in the 0.1 – 1 Hz sub-band (which contains the main components of the EHG signal), a comparison between channels was performed for Flux 90°. Our results showed a higher flux value for channel 1 (data not shown), which could be related to the bipolar electrode configuration, that allows scanning the uterus signal propagation horizontally (through electrodes E2 - E1) and vertically (through Flux 90°). These results agree with those discovered by Diab et al., which confirms that CWT features, such as Flux, describe a physiological pattern of the signal.

Additionally, given that the bandwidth of 1–2 Hz contains the principal interferences caused by the maternal heart rate (7), higher flux was expected for the T group at 90°. This phenomenon was observed in S1F2 (data not shown) and could be related to the proximity held by observant electrode E1 to the maternal heart. Maternal heart influence in channel S1 is amplified, creating a change in power that can be quantifiable by flux analysis.

The main aim of medical research should be at the end to bring discoveries to the clinical field. In this understanding, we homogenized the current methodology aiming to facilitate the eventual transition to clinical practice. For this reason, only records taken around a similar gestation age (e.g., the 31^st^ week of gestation) were included. However, the criterion reduced the sample size, considering the number of available records in the online datasets. Thus, the size of the sample is one of the main limitations of the present study. Future investigations on the preterm labor field should be performed to generate new EHG datasets with more records taken in pregnant women with a similar gestational age.

## 5 Conclusion

The best performance, i.e., 88.52% accuracy, 83.83% sensitivity, and 93.22% specificity in the testing set, 91.30% accuracy in validation, and 93.46% in training was obtained in the 2–3 Hz bands using a Quadratic SVM classification model trained with the EHG features of Medium Frequency, and CWT-derived features such as Flux and Energy, and entropy-based indices. In line with these results, these features exhibited significant differences between term and preterm labor in the EHG signals. Interestingly, CWT features were significantly different in all filter-channel combinations. These differences in the CWT features could be associated with the frequency interference caused by the maternal heart and the placement of electrodes closer to the chest. The main achievement of this study is the determination of key features for preterm birth prediction based on the analysis of EHG. Thus, these results suggest that CWT and novel entropy-based features of EHG could be suitable descriptors for analyzing and understanding the complex nature of preterm labor mechanisms.

## Data availability statement

Publicly available datasets were analyzed in this study. This data can be found here: https://physionet.org/content/tpehgt/1.0.0/ and https://physionet.org/content/tpehgdb/1.0.1/.

## Ethics statement

Ethical review and approval was not required for the study on human participants in accordance with the local legislation and institutional requirements. Written informed consent for participation was not required for this study in accordance with the national legislation and the institutional requirements.

## Author contributions

Conceptualization, JR-L; methodology, HR-M and JM-MO; software, HR-M and JM-MO; validation, HR-M and JM-MO; formal analysis, HR-M and JM-MO; investigation; data curation, HR-M and JM-MO; writing—original draft preparation, YM-P, HR-M, JM-MO, RM-M, and JR-L; writing—review and editing, YM-P, HR-M, JM-MO, RM-M, and JR-L; supervision, JR-L and RM-M; project administration, JR-L; funding acquisition, YM-P. All authors have read and agreed to the published version of the manuscript.

## Footnotes

1 These values represent the PhEn values obtained from S2F3, with a *k*=4. However, a similar tendency in the data was observed throughout other *k* values, and channel selections.
